# Determining Preexponential Factor in Model-Free Kinetic Methods: How and Why?

**DOI:** 10.3390/molecules26113077

**Published:** 2021-05-21

**Authors:** Sergey Vyazovkin

**Affiliations:** Department of Chemistry, University of Alabama at Birmingham, 901 S. 14th Street, Birmingham, AL 35294, USA; vyazovkin@uab.edu

**Keywords:** Arrhenius equation, crosslinking polymerization (curing), decomposition, degradation, liquid and solid state, phase transitions, thermal analysis

## Abstract

The kinetics of thermally stimulated processes in the condensed phase is commonly analyzed by model-free techniques such as isoconversional methods. Oftentimes, this type of analysis is unjustifiably limited to probing the activation energy alone, whereas the preexponential factor remains unexplored. This article calls attention to the importance of determining the preexponential factor as an integral part of model-free kinetic analysis. The use of the compensation effect provides an efficient way of evaluating the preexponential factor for both single- and multi-step kinetics. Many effects observed experimentally as the reaction temperature shifts usually involve changes in both activation energy and preexponential factor and, thus, are better understood by combining both parameters into the rate constant. A technique for establishing the temperature dependence of the rate constant by utilizing the isoconversional values of the activation energy and preexponential factor is explained. It is stressed that that the experimental effects that involve changes in the preexponential factor can be traced to the activation entropy changes that may help in obtaining deeper insights into the process kinetics. The arguments are illustrated by experimental examples.

## 1. Introduction

Kinetic studies of thermally stimulated processes provide a key to understanding the thermal behavior of materials. In the field of condensed phase kinetics, most of such studies are conducted by employing experimental methods such as thermogravimetric analysis (TGA) and differential scanning calorimetry (DSC). The International Confederation for Thermal Analysis and Calorimetry has issued recommendations [1,2] for kinetic analysis of such data. Briefly, proper kinetic analysis can be performed via model-fitting and model-free approaches as long as the calculations are based on simultaneous use of data obtained at multiple temperature programs. Most commonly, it means the usage of data collected at multiple heating (or cooling) rates.

The model-free approach includes primarily the Kissinger method [3] as well as a variety of isoconversional methods [4]. The latter have experienced dramatic growth in popularity over the past two decades [4,5]. An illustrative example is that out of 13 papers in the Special Issue “Thermal Analysis Kinetics for Understanding Materials Behavior” published by Molecules, 10 used isoconversional and other model-free kinetic analyses [6]. At least partially, this is because many of the simpler isoconversional methods can be readily programmed with the common spreadsheets, whereas the model-fitting methods typically require specialized software. A more important difference, though, is that model-fitting methods yield the whole kinetic triplet (i.e., the activation energy, preexponential factor, and reaction model) in a single computational step. Isoconversional methods evaluate the kinetic triplet in several computational steps. This is easily illustrated for a single-step process studied at multiple heating rates. Its kinetics obeys the following rate equation:

(1) $$\frac{d\alpha }{dt}=A\exp\left(\frac{-E}{RT}\right)f\left(\alpha \right)$$

where α is the extent of the reactant conversion, *t* is the time, *f*(*α*) is the reaction model, *T* is the temperature, *R* is the gas constant, and *A* and *E* are the Arrhenius parameters, i.e., the preexponential factor and activation energy, respectively. In this situation, a model-fitting method fits the right hand side of Equation (1) to a set of the rate data obtained at different heating rates. The fitting yields *E*, *A*, and *f*(*α*), all at the same time.

Isoconversional methods rely on the isoconversional derivative of the rate to determine the activation energy as [1,4]:

(2) $$E_{\alpha }=-R{\left[\frac{\partial \ln\left(d\alpha /dt\right)}{\partial T^{-1}}\right]}_{\alpha }$$

where the subscript *α* denotes the value related to specific conversion. The most direct implementation of this approach is the isoconversional method of Friedman [7]. Its basic equation is:

(3) $$\ln{\left(\frac{d\alpha }{dt}\right)}_{\alpha }=\ln\left[A_{\alpha }f\left(\alpha \right)\right]-\frac{E_{\alpha }}{RT_{\alpha }}$$

The application of the method requires one to determine for each conversion the values of the rate and temperature for a set of the heating rates used. Per Equation (3), the left hand side depends linearly on 1/*T_α_*, so that the activation energy is evaluated from the slope of the straight line by means of the standard linear regression. This is the first computational step for isoconversional methods. Evaluating the other two components, i.e., *A* and *f*(*α*), of the kinetic triplet requires additional computational steps, described in detail elsewhere [1,4].

Oftentimes, isoconversional kinetic analysis remains limited to the first step, i.e., to evaluating a dependence of the activation energy on conversion. While incomplete, such analysis can be adequate for certain purposes. For example, the *E_α_* dependence can be used for making kinetic predictions without estimating both the preexponential factor and reaction model [1,8,9,10]. Additionally, this dependence can be parameterized in terms of fundamental kinetic models and used for estimating their parameters [2,4,5,11]. Nevertheless, there are many situations when knowledge of the *E_α_* dependence alone is insufficient for understanding the kinetics. Then, isoconversional analysis must move beyond the first step to evaluate the preexponential factor and reaction model.

Although for complete kinetic analysis both of these components need to be evaluated, the present article does not discuss evaluating the reaction models. This is because the experimentally determined reaction models have very little interpretive value. First, one needs to recognize that the aforementioned computational techniques [1] for estimating the reaction models via isoconversional analysis are suitable only for a single-step process. The latter manifests itself through the absence of significant variation of the isoconversional activation energy with conversion. Variation in *E*_α_ can be ruled as insignificant if within the range *α* = 0.1–0.9 the difference between the maximum and minimum value of *E*_α_ is less than 10–20% of the average *E*_α_ value [2]. Quite frequently, this is not the case. Then, the process cannot be represented by a single model *f*(*α*). Ignoring this point results in computing *f*(*α*) data that cannot and should not be interpreted in terms of the commonly used theoretical *f*(*α*) models. In case of significant variation in *E*_α_ one should use more sophisticated computational techniques, designed specifically for multi-step processes [2]. Second, even if *E*_α_ demonstrates no significant variation so that a process can be considered as a single-step one, the experimentally evaluated *f*(*α*) models still do not permit straightforward interpretation in terms of the mechanisms [12]. Note that all of this is also applicable to the integral models, *g*(*α*).

Instead, this article aims to emphasize the importance of evaluating the preexponential factor in model-free kinetic analysis. The focus is put on the interpretive value of the preexponential factor that is illustrated by several instructive examples. The intention is to demonstrate that determining the preexponential factor alone as well as combining it with the activation energy to evaluate the rate constant can help in obtaining deeper insights into the process kinetics.

## 2. How to Determine the Preexponential Factor

There are several methods of estimating the preexponential factor in model-free and isoconversional calculations. Fundamentally, they can be reduced to two approaches: model-based and model-free. Friedman, who proposed the earliest isoconversional method for kinetic analysis of nonisothermal data, also proposed a model-based way of evaluating the preexponential factor [7]. The evaluation makes use of the fact that the application of Equation (3) produces not only a dependence of *E*_α_ on α but a dependence of ln[*A_α_f*(*α*)] on α as well. Then, assuming a particular form of the reaction model, e.g., the reaction-order model *f*(*α*) = (1 − α)*^n^*, one can readily use the ln[*A_α_f*(*α*)] dependence to estimate both ln*A_α_* and *n* [7]. There are more advanced variants of the model-based approach that are not limited to the reaction-order model. Some of them are discussed in the ICTAC recommendations [1]. While different, all model-based techniques share a common theme. They identify the reaction model to evaluate the preexponential factor. This approach works perfectly well provided that the process analyzed can be considered as a single-step one—in other words, when *E*_α_ does not demonstrate significant variation with *α*.

**Figure 1:**
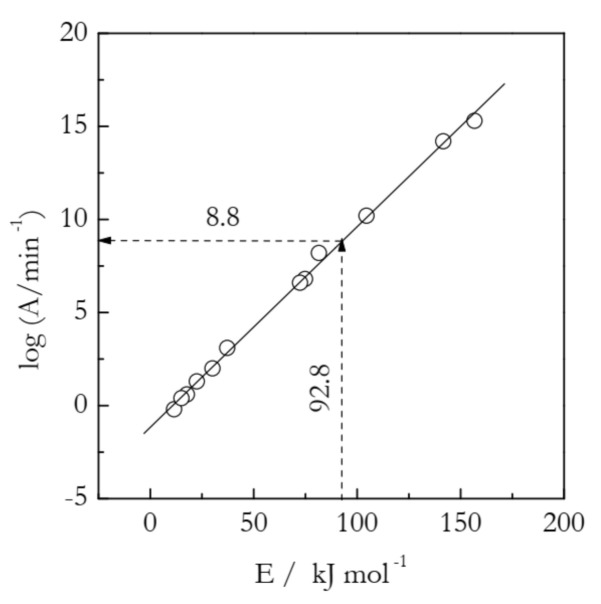
Compensation effect (solid line) for thermal decomposition of ammonium nitrate. Circles are individual ln*A_i_* and *E_i_* values calculated for 12 models from Table 1. A model-free estimate of ln*A* (8.8) is determined by substituting a model-free value of *E* (92.8 kJ mol^−1^) into the compensation effect Equation (5). Adapted with permission from Vyazovkin et al. [13]. Copyright 2001 ACS.

If *E*_α_ varies significantly with *α*, i.e., when the process is a multi-step one, the preexponential factor can be determined via a model-free approach. The idea is to make use of the so-called compensation effect that takes the following form:

(4) $$\log A_{i}=aE_{i}+b$$

where the subscript *i* denotes *A* and *E* determined experimentally when using a particular *f_i_*(*α*)-model to fit the rate data obtained at a single heating rate. This type of fit is well-known to produce *A_i_* and *E_i_* that depend strongly on the choice of *f_i_*(*α*) in the differential methods or of *g_i_*(*α*) in the integral methods. An example of the compensation effect is illustrated in Table 1 and Figure 1 for the thermal decomposition of ammonium nitrate analyzed by an integral method [13].

It is seen that for a set of 12 *g_i_*(*α*) models presented in Table 1, the log*A_i_* and *E_i_* values vary in a very broad range. Yet, they all fit almost perfectly into a straight line:

(5) $$\log A_{i}=0.108E_{i}-1.17$$

The existence of the compensation effect suggests that all log*A* and *E* pairs, be they correct or not, are linearly correlated. It means that knowing a model-independent value of *E* should allow one to plug it into the compensation effect Equation (4) and estimate the respective value of log*A*. This is the idea of the model-free approach to estimating the preexponential factor [14,15]. Simulations by Vyazovkin et al. have demonstrated the high accuracy of this approach for both single- [14] and for multi- [15] step kinetics.

If the *E*_α_ values do not vary significantly with *α*, their substitution into Equation (4) will yield the log*A*_α_ values that also are nearly independent of α. In this situation, the *E*_α_ values can be simply replaced with a single mean value, the substitution of which into Equation (4) will yield a single mean value of log*A*_α_. This example is presented in Figure 1. For the decomposition of ammonium nitrate, *E*_α_ is practically independent of α [13]. The mean value is 92.8 kJ mol^−1^. Its substitution into Equation (5) yields log*A* = 8.8.

In connection with the ammonium nitrate example, a warning must be given regarding another procedure for estimating the preexponential factor. In it, a model-free estimate of *E* is obtained, and its value is compared against the activation energies estimated by using various reaction models. Then, one selects the model that gives the closest value *E* and uses the respective value of the preexponential factor as a proper estimate. This procedure is clearly inferior to the one based on utilizing the compensation effect for two reasons. First, it is equivalent to picking one point on the compensation line and, as seen in Figure 1, the individual points do fluctuate around the line. These fluctuations will introduce an error in the log*A* estimate even if there is a close match between the model-free and model-based values of *E*. On the other hand, the use of the compensation line eliminates all individual fluctuations and, thus, the error associated with them. Second, it is quite common that there is no model that yields a closely matching value of *E*. An instructive example is found in Table 1. There are no *E* values that closely match the model-free value 92.8 kJ mol^−1^. The two closest values are 81.5 and 104.5 kJ mol^−1^. Their respective log*A* values are 8.2 and 10.2, neither of which is quite accurate.

Most important for our purpose is that the afore-described model-free approach provides accurate estimates for the log*A*_α_ value in the case of multi-step kinetics. This has been originally established by Vyazovkin and Linert [15] and also demonstrated recently by Sbirrazzuoli, [16] who has made significant contributions [17] in perfecting this approach. As stated earlier, the multi-step kinetics is identified by significant variation of *E*_α_ with *α*. In this case, the *E*_α_ dependence cannot be replaced with the mean value. Then, substitution of the *E*_α_ values into Equation (4) yields a dependence of log*A*_α_ on *α* that, as shown further, can be used for obtaining important insights as well as for estimating the temperature dependence of the rate constant.

It should be stressed that the log*A*_α_ values can be estimated from Equation (4) by substituting the *E*_α_ values determined by any isoconversional method. As seen from a few recent examples [18,19,20,21] of estimating log*A*_α_ via the compensation effect, one typically uses *E*_α_ determined by the most popular isoconversional methods such as the integral method of Ozawa [22] and Flynn-Wall [23] and of Kissinger-Akahira-Sunose [24] or as the differential method of Friedman [7]. With respect to this, it should be reminded that these integral methods, also known [4,25] as stiff integral methods, introduce a systematic error into the *E*_α_ value when it varies significantly with α [26]. This error naturally propagates into a systematic error in the log*A*_α_ value. To eliminate this error, one should use flexible integral methods [4,25]. Popular representatives of these method are the isoconversional method of Vyazovkin [26] or Ortega [27]. Alternatively, one can use the differential method of Friedman to avoid the systematic error in *E*_α_ and, thus, in log*A*_α_. In addition, as discussed by Sbirrazzuoli, [17] the accuracy of the log*A*_α_ value is improved by using more accurate methods for estimating log*A*_i_ and *E*_i_ that are inserted in Equation (4) for determining the parameters *a* and *b*.

Kissinger did not propose a way of determining the preexponential factor in his model-free method. He did propose a method of estimating the reaction order from the so-called shape index, *S* as $n=1.26S^{0.5}$, where *S* is evaluated experimentally from the rate peak. Balarin [28] has found this relationship inaccurate and suggested an alternative expression, $n=1.54S^{1.18}$. As far as estimating the preexponential factor, one needs first to recognize that unlike isoconversional methods, the Kissinger method typically fails to detect the multi-step kinetics [29]. In other words, it treats all processes as single-step kinetics. For that reason, the preexponential factor in this method can be estimated by means of model-based techniques suitable for single-step kinetics [1] as well as with the aid of the model-free approach described above.

## 3. Why to Determine the Preexponential Factor

### 3.1. Preexponential Factor for Understanding Reaction Temperature Shifts

**Figure 2:**
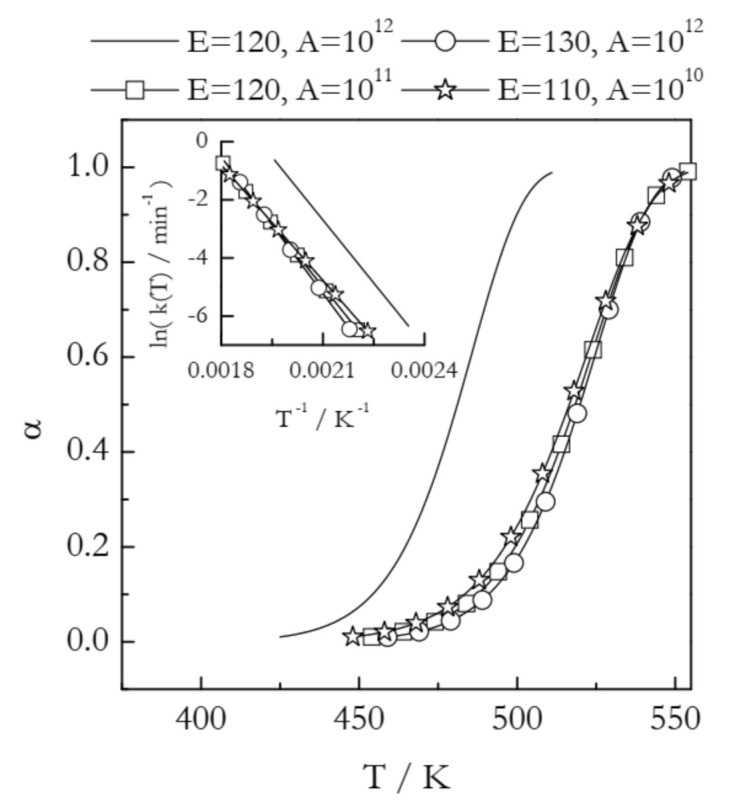
Kinetic curves simulated at the heating rate 2 K min^−1^ for a first order reaction having different values of the activation energy (*E* in kJ mol^−1^) and preexponential factor (*A* in min^−1^). The inset shows respective Arrhenius plots.

Materials are routinely modified to change their thermal behavior. Some applications may require lowering the reaction temperature, whereas others may need the reaction temperature to be increased. Such changes are readily seen in nonisothermal TGA or DSC curves as shifts to either lower or higher temperature. For example, Figure 2 illustrates the situation when the modification of a compound causes an increase in its thermal stability. This is best seen as a shift in the conversion vs. temperature curves. All four curves presented in Figure 2 have been simulated for the reaction model of first-order. The solid line represents a process with *E* = 120 kJ mol^−1^ and *A* = 10^12^ min^−1^. The three other curves appear at a temperature about 40 K higher and, thus, represent increased thermal stability. From the kinetic standpoint, an increase in thermal stability is associated with the deceleration of the process. The most common explanation for deceleration is an increase in the activation energy. Overcoming a larger energy barrier naturally requires increasing the kinetic energy of the reactant molecules, which is accomplished by increasing the temperature. In Figure 2, this effect is represented by the curve with circles (*E* = 130 kJ mol^−1^ and *A* = 10^12^ min^−1^). Indeed, an increase in *E* without a change in *A* decelerates the process by shifting its range to higher temperature. This type of effect would be easily detected by estimating the activation energy with the aid of any model-free method.

However, Figure 2 depicts two more examples of very similar temperature shifts. The curve with squares (*E* = 120 kJ mol^−1^ and *A* = 10^11^ min^−1^) demonstrates that deceleration and a shift to higher temperature can occur at the expense of a decrease in *A* without any changes in *E*. While uncommon, such a case has been observed experimentally in a study of the effect of inert gas pressure on the kinetics of reversible decomposition [30]. Furthermore, as seen from the curve with stars (*E* = 110 kJ mol^−1^ and *A* = 10^10^ min^−1^), deceleration and a shift to higher temperature can happen even when *E* decreases if *A* undergoes a significant decrease as well. Needless to say that in such situations estimating the activation energy alone is not able to help in identifying the reason behind the observed shift to higher temperature, i.e., an increase in thermal stability. Understanding the observed effect necessarily requires estimating the preexponential factor.

Note that changes in thermal behavior rarely entail a change in only one of the Arrhenius parameters. Typically, both *E* and *A* change simultaneously. This implies that understanding the shifts in the reaction temperature generally requires consideration of the combined effect of *E* and *A*. Such an effect is accounted for in the rate constant, *k*(*T*). The significance of this parameter is that it provides a measure of the reactivity. The rate constant depends on temperature in accord with the Arrhenius equation:

(6) $$k\left(T\right)=A\exp\left(\frac{-E}{RT}\right)$$

Plugging experimentally determined *A* and *E* in Equation (6) and considering the *k*(*T*) values in the corresponding temperature regions permits visualizing the combined effect of changes in both Arrhenius parameters. Figure 2 (inset) shows the Arrhenius plots for the four examples discussed above. Comparison of the *k*(*T*) values at the same temperature clearly shows that all three shifts to higher reaction temperature represent a nearly identical decrease in the reactivity of the system considered. Note that in practical situations when the kinetics is multi-step and, thus, both *E*_α_ and *A*_α_ vary with α, linking temperature shifts to either parameter becomes especially difficult [31]. Thus, combining *E*_α_ and *A*_α_ into the rate constant and analyzing the resulting Arrhenius plot provides an effective solution to the problem.

Building the Arrhenius plots is trivial in the case of the single-step kinetics. As already stated, in such a case *E*_α_ and *A*_α_ do not practically vary with α so that they can be replaced with the mean values. The latter just need to be inserted in Equation (6). Creating the Arrhenius plots for multi-step kinetics, i.e., when both *E*_α_ and *A*_α_ vary significantly with α, is a bit trickier. An appropriate procedure for calculating the Arrhenius plots for multi-step kinetics has been proposed by Liavitskaya et al. [31]. The essential step in this procedure is converting the dependencies of *E*_α_ and *A*_α_ on α to the dependencies on temperature. Recall that in the isoconversional calculations each *E*_α_ is estimated for a given value of α by using several values of *T*_α_, which are the temperatures of reaching this α at different heating rates. That is, each value of *E*_α_ is associated with several *T*_α_ values that can be replaced with their mean value. Then, replacing α with the respective mean *T*_α_ in the dependencies of *E*_α_ and *A*_α_ on α yields the dependencies of *E*_α_ and *A*_α_ on temperature [11]. The latter can then be substituted into Equation (7):

(7) $$\ln k\left(T_{\alpha }\right)=\ln A_{\alpha }-\frac{E_{\alpha }}{RT_{\alpha }}$$

to yield the Arrhenius plots for the multi-step kinetics.

**Figure 3:**
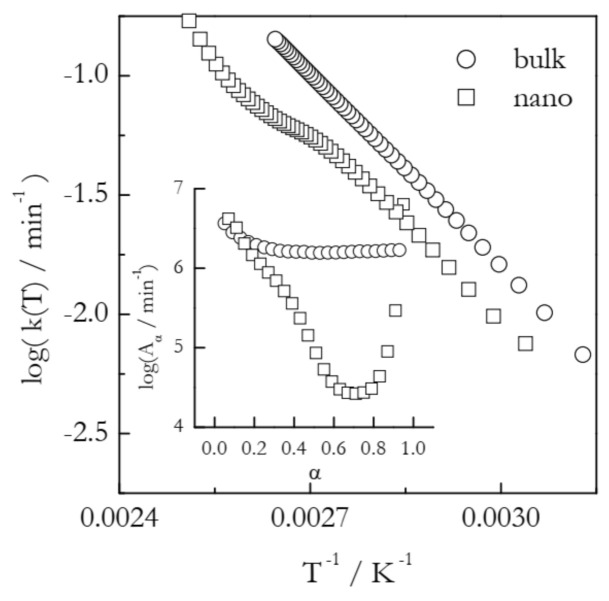
Dependencies of the preexponential factor on conversion and respective Arrhenius plots for vaporization of *n*-decane from bulk (circles) and nanopores (squares). Adapted with permission from Ekawa et al. [32]. Copyright 2021 Elsevier.

An example of the Arrhenius plots built according to Equation (7) is displayed in Figure 3. The plots represent the process of vaporization of *n*-decane from bulk and 7 nm alumina nanopores [32]. In the nanopores, vaporization is markedly decelerated as detected by a shift of the TGA curves to a higher temperature. Isoconversional calculations for bulk vaporization yield the *E_α_* values that are practically independent of α and average at ~56 kJ mol^−1^, which is the value of the vaporization enthalpy of *n*-decane. The *E*_α_ values for vaporization from the nanopores drop from that value down to ~40 kJ mol^−1^. On the other hand, a decrease in *E*_α_ is accompanied by a ~2 orders of magnitude decrease in *A*_α_ (Figure 3, inset). It is worth mentioning that when *E*_α_ varies significantly with α, i.e., when the kinetics is multi-step, the Arrhenius plots should generally be nonlinear [11]. In the same way, practically invariable *E*_α_ representative of single-step kinetics should give rise to linear Arrhenius plots. This is exactly what one can see in Figure 3: a linear plot for vaporization from bulk and a nonlinear one for vaporization from the nanopores. In any case, the obtained Arrhenius plots indicate that at any given temperature within the experimental range, vaporization from the nanopores is characterized by a smaller rate constant. It means that the observed effect occurs because possible acceleration associated with a decrease in *E*_α_ is completely outweighed by deceleration due to a decrease in *A*_α_.

Of course, the above example is not the only case of the benefit provided by analysis of the Arrhenius plots constructed from the *E*_α_ and *A*_α_ dependencies. As mentioned earlier, Liavitskaya et al. [31] have proposed the aforementioned procedure as a means to understand the temperature shifts observed experimentally in the situation when both *E*_α_ and *A*_α_ varied with conversion. This has occurred in a study of the thermal decomposition of malonic acid dissolved in three different polymeric matrixes: poly(vinylpyrrolidone), poly(methyl methacrylate), and poly(vinyl acetate). Although comparison of the individual Arrhenius parameters for these systems does not demonstrate any simple trend, a clear-cut trend is seen in the Arrhenius plots that reveal a significant acceleration of decomposition in the poly(vinylpyrrolidone) matrix. Likewise, this procedure has been instrumental in obtaining insights into the effect of the poly(vinylpyrrolidone) matrix on the thermal stability of such drugs as indomethacin, felodipine, and nifedipine [33]. It has also helped to visualize the saturation in the increase in the thermal stability of the solid dispersions of indomethacin in poly(vinylpyrrolidone) that occurs at a specific drug to polymer ratio [34]. Other recent examples of the beneficial use of the proposed procedure for calculating the Arrhenius plots include: evaluating the thermal stabilities of terpolymers of carbon dioxide, propylene oxide, and cyclohexene oxide [18]; probing a catalytic effect of nanosized zinc and titanium oxides on the thermal degradation of poly(lactic acid) [35]; assessing the reactivity toward the polymerization of aryl cyanates with different bridging fragments [36]; exploring the effect of ionic liquid treatment on the oxidation kinetics of coal [37]; studying the effect of milling on the oxidation kinetics of aluminum–boron systems [38]; gauging the acceleration of the cyanate ester polymerization in hydrophilic nanopores of silica colloidal crystals [39]; and comparing the thermal decomposition kinetics of an ionic liquid under nitrogen and air [40].

### 3.2. Entropic Interpretation of Changes in the Preexponential Factor

The temperature dependence of the rate constant is traditionally described by the Arrhenius Equation (6). The activated complex theory suggests a rather similar form for this dependence [41]:

(8) $$k\left(T\right)=\frac{k_{B}T}{h}\exp\left(\frac{\Delta S^{\neq }}{R}\right)\exp\left(\frac{-\Delta H^{\neq }}{RT}\right)$$

where *k*_B_ is the Boltzmann constant, *h* is the Planck constant, and Δ*S*^≠^ and Δ*H*^≠^, respectively, are the entropy and enthalpy of activation. Δ*S*^≠^ represents the difference between the entropy of the activated complex (S_AC_) and the entropy of reactants (S_R_). Δ*H*^≠^ represents a similar difference in the enthalpies. Δ*H*^≠^ in Equation (8) is smaller than *E* in Equation (6) by one or two *RT* values for mono- or bimolecular reactions, respectively. However, this difference usually does not exceed the typical 5–10% uncertainty in experimental activation energy and, thus, can be ignored. Replacing Δ*H*^≠^ with *E* in Equation (8) and comparing it to Equation (6) allows one to define the preexponential factor in the Arrhenius equation as:

(9) $$A=\frac{k_{B}T}{h}\exp\left(\frac{\Delta S^{\neq }}{R}\right)$$

Alternatively, the preexponential factor can be called the “frequency factor” because the term *k*_B_*T*/*h* represents a frequency, at which the activated complex at the top of the energy barrier decomposes into the reaction products [41]. The importance of the Δ*S*^≠^ term in Equation (9) is that it contributes to the shift in equilibrium between the reactants and activated complexes via the usual thermodynamic relationship:

(10) $$\Delta G^{\neq }=\Delta H^{\neq }-T\Delta S^{\neq }$$

where Δ*G*^≠^ is the free energy of the activated complex formation. An increase in Δ*S*^≠^ lowers Δ*G*^≠^, thereby shifting the equilibrium towards the formation of the activated complexes. To put it simply, the Δ*S*^≠^ term is responsible for the number of the activated complexes formed. All things considered, the preexponential factor, as defined in Equation (9), can be thought of as the intensity of the reaction attempts.

For bimolecular reactions, Δ*S*^≠^ is typically negative. This results from the diminishing of the degrees of freedom for the activated complex relative to the reactants. For example, if a reaction system of two molecules has six translational and six rotational degrees of freedom, there only are three translational and three rotational degrees of freedom for the activated complex formed from these two molecules. For monomolecular reactions, the situation is more complex and the Δ*S*^≠^ values can take on both positive and negative values. Yet, the former seem more common. The techniques for theoretical estimation of the activation entropies have been discussed in detail elsewhere [42].

**Figure 4:**
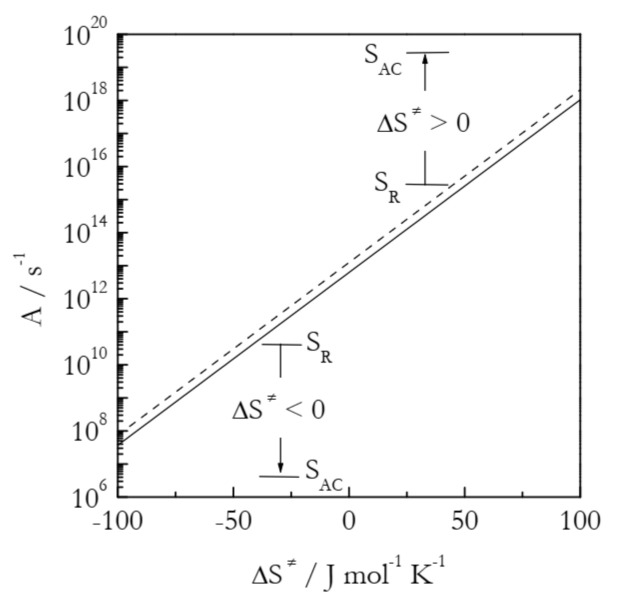
Preexponential factor as a function of the activation entropy at temperatures 300 K (solid line) and 600 K (dashed line). S_AC_ and S_R_ are the entropies of the activated complex and reactants, respectively.

As seen in Figure 4, the preexponential factor is not affected much by temperature, which is the reason why its temperature dependence is usually ignored within relatively narrow temperature regions used experimentally. However, it depends quite strongly on the activation entropy. The trend is simple: an increase in the activation entropy gives rise to an increase in the preexponential factor and, thus, to acceleration. It is worth noting that an increase in Δ*S*^≠^ represents different situations for positive and negative activation entropies. For the former, an increase in Δ*S*^≠^ corresponds to an increase in the gap between S_AC_ and S_R_. In the case of negative Δ*S*^≠^, the larger value means a smaller negative number, i.e., a decrease in the gap between S_AC_ and S_R_ (see Figure 4).

The methods of determining the preexponential factor discussed earlier can be employed for estimating the activation entropy as:

(11) $$\Delta S^{\neq }=R\ln\left(\frac{Ah}{k_{B}T}\right)\;$$

This parameter can provide certain insights into the experimentally observed kinetic effects such as the reaction temperature shifts. To illustrate this point, we consider two examples that involve the cases of the positive and negative activation entropy.

**Figure 5:**
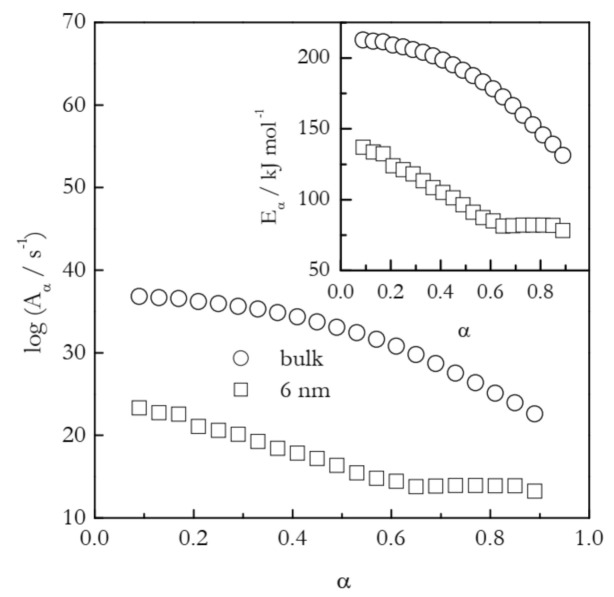
Isoconversional values of the activation energy and preexponential factor for melting of gelatin gel in bulk (circles) and 6 nm nanopores (squares). Adapted with permission from Prado et al. [43]. Copyright 2016 PCCP Owner Societies.

The first example deals with the temperature shift in the case of gelatin gel melting [43]. This physical gel is crosslinked by hydrogen bonds. The latter support the self-assembly of the polypeptide chains into the network junction points that control the thermal stability of the gel. Gel melting essentially is the process of the dissociation of hydrogen bonds that hold together the junction points. Figure 5 shows the dependencies of *E*_α_ and log*A_α_* calculated by means of the Vyazovkin isoconversional method [26] for the melting of the gel in bulk and inside 6 nm silica pores. The process in the nanopores occurs at about 10 K larger temperature than in bulk [43]. This suggests that the confinement of the gel to the nanopores decelerates the rate of its melting. Nevertheless, the activation energy for the process in nanopores is significantly smaller than the value determined for bulk melting. Clearly, the effect arises from the fact that the preexponential factor for the nanoconfined process is dramatically smaller than that for the bulk process.

The application of Equation (11) to the values of *A_α_* presented in Figure 5 affords estimating the activation entropies for both processes. Naturally, a variable value of A_α_ should give rise to a variable value of Δ*S*^≠^. Indeed, for bulk Δ*S*^≠^ decreases from ~400 to 195 J mol^−1^ K^−1^ and for nanopores from 210 to 20 J mol^−1^ K^−1^. That is, the activation entropy for the nanoconfined process is much smaller than for the bulk melting. Since Δ*S*^≠^ is positive, its decrease is associated with decreasing the gap between S_AC_ and S_R_ (see Figure 4). Considering the values of S_R_ and S_AC_ separately, one can expect nanoconfinement to diminish the aforementioned gap by either increasing S_R_ or decreasing S_AC_. Taking into account that nanoconfinement generally constrains the molecular motion, it is reasonable to assume that the effect observed is due to a decrease in S_AC_. It can, therefore, be concluded that nanoconfinement restricts the mobility of the activated complex and, thus, stabilizes it against hydrogen bond breaking. This conclusion is in agreement with the original study that has demonstrated [43] that nanoconfinement promotes the restoration of the broken hydrogen bonds. Overall, both activation entropy and preexponential factor drop, giving rise to the deceleration of the process under nanoconfinement.

The second example is concerned with the effect of nanoconfinement on the trimerization of potassium and rubidium dicyanamide (KDCA and RbDCA, respectively) [44]. The process in 4 and 30 nm pores of silica reveals significant acceleration detected as the lowering of the reaction temperature by 20–50 K relative to the bulk process. Table 2 collects the Arrhenius parameters for both bulk and nanoconfined processes. The values have been estimated via the Kissinger method as discussed above. It is seen that the process in nanopores has a markedly larger activation energy than that in bulk. Therefore, the observed acceleration should be linked to the dramatic increase in the preexponential factor. Table 2 also presents the activation entropies estimated by plugging the *A* values in Equation (11). The nanoconfined process consistently demonstrates significantly larger Δ*S*^≠^ values (i.e., significantly smaller negative numbers). The activation entropies are negative, as should be expected for a bimolecular process. As Δ*S*^≠^ is negative, its increase for the nanoconfined process is associated with diminishing the gap between S_AC_ and S_R_ (see Figure 4). The gap can be decreased by either lowering S_R_ or raising S_AC_. As already mentioned, nanoconfinement generally suppresses molecular mobility. Thus, it should be expected that Δ*S*^≠^ rises because of the drop in S_R_. As suggested in the original study [44], the reactant molecules appear to undergo some ordering along the nanoconfining surface, attaining a preferential reactive situation. In all, the activation entropy and preexponential factor rise, causing the nanoconfined process to accelerate.

As a final point, a brief word of caution is necessary in regard to the entropic interpretations of the preexponential factor of the condensed phase reactions. The underlying theory is the theory of an elementary reaction act occurring in the absence of a reaction medium. The condensed phase reactions tend to occur as a complex interplay of various elementary reaction acts oftentimes complicated by diffusion. For that reason, the activation energies or the preexponential factors, determined experimentally by such methods as DSC or TGA, generally have a meaning of effective (or overall, global, apparent, etc.) parameters [11]. The effective activation energy and preexponential factor typically represent more than one elementary reaction act. The effective nature of the activation energy and preexponential factor naturally propagates into other parameters derived from them, such as Δ*S*^≠^, Δ*H*^≠^, and Δ*G*^≠^. Therefore, their interpretation in terms of elementary act theories must be conducted sensibly. In particular, it is sensible to limit the entropic interpretations of the preexponential factor to semi-quantitative trends. As shown in the above examples, identifying such trends can be sufficient in obtaining deeper kinetic insights.

## 4. Conclusions

This article has highlighted the importance of determining the preexponential factor as a part of model-free kinetic analysis. The emphasis has been on using a model-free way of estimating the preexponential factor because it is suitable for both single- and multi- step kinetics, i.e., for cases when the isoconversional activation energy does not practically vary with conversion and when it varies with conversion significantly. It has been stressed that experimentally observed effects such as reaction temperature shifts are typically associated with changes in both the activation energy and preexponential factor. Thus, they are better understood by quantifying the joint effect of both parameters in the form of the rate constant. A technique for building Arrhenius plots from the isoconversional values of the activation energy and preexponential factor has been discussed. Lastly, attention has been drawn to the fact that the experimental effects that entail changes in the preexponential factor can be interpreted in terms of the activation entropy changes, thereby providing deeper insights into the process kinetics. It is hoped that this brief article will inspire more workers to extend their model-free kinetic analyses beyond exclusively estimating the activation energy.

## Figures and Tables

**Table 1:** Activation energies for the thermal decomposition of ammonium nitrate determined using 12 different models. Adapted with permission from Vyazovkin et al. [13]. Copyright 2001 ACS.

i	Reaction Model	g_i_(α)	E_i_/kJ mol^−1^	log(A_i_/min^−1^)
1	power law	α^1/4^	11.5	−0.2
2	power law	α^1/3^	17.7	0.6
3	power law	α^1/2^	30.1	2.0
4	power law	α^3/2^	104.5	10.2
5	one-dimensional diffusion	α^2^	141.6	14.2
6	Mampel (first order)	−ln(1 − α)	81.5	8.2
7	Avrami–Erofeev	[−ln(1 − α)]^1/4^	15.1	0.4
8	Avrami–Erofeev	[−ln(1 − α)]^1/3^	22.5	1.3
9	Avrami–Erofeev	[−ln(1 − α)]^1/2^	37.2	3.1
10	three-dimensional diffusion	[1 − (1 − α)^1/3^]^2^	156.7	15.3
11	contracting sphere	1 − (1 − α)^1/3^	74.8	6.8
12	contracting cylinder	1 − (1 − α)^1/2^	72.4	6.6

**Table 2:** Kinetic parameters for trimerization of KDCA and RbDCA in the nanopores and bulk. Reproduced with permission from Yancey and Vyazovkin [44]. Copyright 2015 PCCP Owner Societies.

System	*E*/kJ mol^−1^	log(*A*/s^−1^)	Δ*S*^≠^/J mol^−1^ K^−1^
Bulk KDCA	54.5 ± 1.8	2.4 ± 0.1	−206
30 nm KDCA	93.3 ± 6.8	5.7 ± 0.1	−142
4 nm KDCA	101.6 ± 7.4	6.8 ± 0.2	−121
Bulk RbDCA	50.1 ± 4.5	2.4 ± 0.1	−205
30 nm RbDCA	92.3 ± 9.4	6.8 ± 0.1	−120
4 nm RbDCA	91.9 ± 5.3	6.7 ± 0.1	−122

## Data Availability

Not applicable.

## Sample Availability

Samples of the compounds are not available from the authors.
